# Mask‐related anxiety and distress during radiation therapy for head and neck cancer

**DOI:** 10.1002/jmrs.695

**Published:** 2023-06-16

**Authors:** Haryana M. Dhillon, Georgia K. B. Halkett

**Affiliations:** ^1^ Faculty of Science, School of Psychology, Psycho‐Oncology Cooperative Research Group University of Sydney Sydney New South Wales Australia; ^2^ Centre for Medical Psychology & Evidence‐Based Decision‐Making Sydney New South Wales Australia; ^3^ Curtin School of Nursing/Curtin Health Innovation Research Institute (CHIRI), Faculty of Health Sciences Curtin University Bentley Western Australia Australia

## Abstract

Some patients experience mask‐related anxiety and distress when undergoing radiation therapy for head and neck cancer. Building on the paper by Forbes et al (doi.org/10.1002/jmrs.658) this editorial discusses techniques to implement to improve the patient experience through education and support.

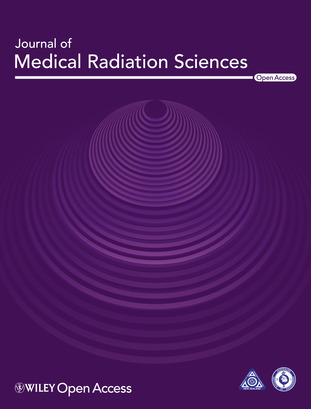

Radiation Therapy (RT) is a key treatment modality for people diagnosed with head and neck cancer (HNC). Due to the anatomy of the head and neck region, precision in treatment is critical. To ensure safety, patient use of an immobilisation mask is standard practice, but its use can be distressing for individuals. Recognising the, sometimes, high levels of anxiety and the variable trajectory of anxiety experienced by people with HNC during their RT there has been a growing body of evidence describing the patient experience using a range of research methodologies.^1^, ^2^, ^3^, ^4^ Forbes et al.^5^ report on a small qualitative exploration of patient experience of the immobilisation mask and strategies to address anxiety. Five patients who self‐reported mask anxiety during treatment participated in semi‐structured interviews. The six main themes discussed included: triggers of anxiety; adjusting to radiation therapy; education about the mask; coping; motivation and improving the patient experience. Recommendations made by Forbes et al.^5^ include increased communication from health professionals, delivery of visual information opportunity to interact with masks prior to treatment, and providing patients with the opportunity to control music played in the room. In this editorial, we further consider how we can support patients to reduce their mask‐related anxiety and distress. Our suggestions focus on language, patient preparation and digital innovations that are currently being trialled.

## Language

Using the term ‘immobilisation’ mask is likely to contribute to individuals' anxiety. A very simple, easily implementable change in practice would be to reframe the language used. Renaming immobilisation masks as ‘safety’ masks moves from imagery of restraint to something more positive, as suggested by HNC survivors and advocates. Such a change helps patients understand the mask is keeping them safe during their treatment. While this is unlikely to transform the experience for all patients, it is likely to contribute toward lower anxiety overall during RT treatment via a low‐cost change in practice. This rephrasing of language is captured by consumer advocate Julie McCrossin in her work with the Radiation Oncology Targeting Cancer Campaign.^6^

## Patient preparedness

A key finding of Forbes et al.^5^ is lack of information about and preparation for use of the mask, consistent with other qualitative studies in this population.^2^, ^4^ It is surprising given what is known about the importance of preparation of patients for treatment, especially treatments considered aversive, that we are not doing better in preparing patients for use of a safety mask for HNC treatment. Radiation therapists have an important role to play in communicating with patients, providing information and support, and preparing them for radiation treatment.^7^, ^8^ Given radiation therapists see patients daily during treatment, they are well positioned to develop supportive relationships with potential to identify and address psychosocial concerns, referring patients to psycho‐oncology staff where relevant.

Halkett et al.^9^ previously demonstrated the effectiveness of RT Prepare, an intervention designed to improve the preparation radiation therapists give to people starting treatment for breast cancer. In the RT Prepare intervention, radiation therapists engaged patients in two consultations (prior to treatment planning and treatment) to improve patient preparation and reduce patient distress. Radiation therapists delivering the intervention participated in communication skills training which focused on providing sensory and procedure information relating to radiation therapy and addressing patients' pre‐treatment anxiety. Overall, the RT Prepare study demonstrated it is feasible to deliver communication skills training and improved education materials to prepare people with breast cancer for radiation treatment, reducing their distress.^9^ Radiation therapists participating in communication skills training reported the most valuable components of the RT Prepare workshops were role plays with an actor; receiving feedback on their skills; and, the skills and knowledge gained about improving their communication skills.^10^ RT Prepare was inexpensive to deliver compared to other psycho‐oncology interventions.^11^ This is clearly an intervention with potential for expansion and adaptation to other tumour groups where distress can be expected and severe.

Additionally, there is low recognition of the prevalence and severity of patient anxiety before and during radiation treatment. Using written case study vignettes, Elsner et al.^12^ demonstrated radiation therapists are able to detect anxiety and endorse management strategies appropriate to their clinical setting. Burns et al.^1^ found radiation therapists consistently assessed patients anxiety levels lower than the levels reported by patients themselves. This suggests the need for routine screening for distress during the treatment period to ensure patients experiencing anxiety are identified and provided appropriately tailored support to reduce their anxiety. One such approach to distress screening in clinical practice involved the implementation of a clinical pathway for managing anxiety and depression.^13^ The Psycho‐Oncology Cooperative Research Group (POCOG) implemented distress screening supported by tailored clinical pathways for patient management across 12 cancer services in New South Wales, Australia.^14^ Furthermore, PROMPT‐Care, a symptom screening system, has been implemented in some hospitals in NSW demonstrating a reduction in use of emergency department for people in treatment.^15^

Arnold et al. built on this work by exploring the impact of communication skills training for radiation therapists combined with implementing distress screening in routine practice.^16^, ^17^ Communication skills training increased radiation therapist confidence, knowledge, and attitudes toward their role in providing psychosocial care to patients.^16^ Additionally, more patients underwent screening for distress after radiation therapists participated in communication skills training.^16^

Digital technology can also be used to improve patient education about radiation therapy. Studies are currently in progress trialling the use of virtual reality‐based education for patients receiving radiation therapy.^18^, ^19^ As highlighted by Forbes et al.,^5^ people diagnosed with head and neck cancer are likely to benefit from visual information about treatment and the opportunity to interact with the mask prior to treatment.

## Digital innovations designed to improve treatment experience

Innovative strategies, such as movement tracking and real‐time adjustment to RT delivery, which aim to remove the need for safety masks are in development and under investigation. The SMART protocol is a pilot feasibility study prospectively assessing patient acceptance, state anxiety, and patient experience in people currently being treatment with radiation therapy for head and neck cancer.^20^ However, it will be sometime before the accuracy and safety of these strategies is known, and they are potentially able to be implemented in clinical practice. It is critical that any changes in treatment delivery also consider the impact on patients. For example, removal of the mask may increase patient anxiety and fear of treatment due to the absence of any device to assist them in remaining still.

## Conclusion

By acknowledging that patients experience mask‐related anxiety and distress, much can be done to improve their experience and reduce this distress. Radiation therapists have a key role to play because they treat patients on a daily basis and observe how patients respond to their mask and treatment procedures. While future research is needed to evaluate interventions to prepare and support people with head and neck cancer treatment, it is critical hospital systems incorporate existing evidence‐based approaches to screening and managing distress.

## Conflict of interest

The author declares that they have no competing interests.

## Data Availability

Data sharing not applicable to this article as no datasets were generated or analysed during the current study.

## References

[1] Burns M , Campbell R , French S , et al. Trajectory of anxiety related to radiation therapy mask immobilization and treatment delivery in head and neck cancer and radiation therapists' ability to detect this anxiety. Adv Radiat Oncol 2022; 7: 100967. 36148368 10.1016/j.adro.2022.100967 PMC9486416

[2] Nixon JL , Cartmill B , Turner J , et al. Exploring the prevalence and experience of mask anxiety for the person with head and neck cancer undergoing radiotherapy. J Med Radiat Sci 2018; 65: 282–90. 30378282 10.1002/jmrs.308 PMC6275267

[3] Nixon JL , Brown B , Pigott AE , et al. A prospective examination of mask anxiety during radiotherapy for head and neck cancer and patient perceptions of management strategies. J Med Radiat Sci 2019; 66: 184–90. 31343118 10.1002/jmrs.346 PMC6745384

[4] Keast R , Sundaresan P , Burns M , Butow PN , Dhillon HM . Exploring head and neck cancer patients' experiences with radiation therapy immobilisation masks: A qualitative study. Eur J Cancer Care (Engl) 2020; 29: e13215. 31883285 10.1111/ecc.13215

[5] Forbes E , Clover K , Baker AL , Britton B , Carlson M , McCarter K . 'Having the mask on didn't worry me until … they clamped my head down so I wouldn't move': A qualitative study exploring anxiety in patients with head and neck cancer during radiation therapy. J Med Radiat Sci 2023: 1–9. 10.1002/jmrs.658 PMC10500108 36724485

[6] Radiation Oncology Targeting Cancer . Julie's Story: Radiation Oncology Targeting Cancer. 2015 [cited 2023 May 10]. Available from: https://www.targetingcancer.com.au/our-stories/Julies-story/.

[7] Elsner K , Naehrig D , Halkett G , Dhillon H . Reduced patient anxiety as a result of radiation therapist‐ led psychosocial support: a systematic review. J Med Radiat Sci 2017; 64: 220–31. 28160448 10.1002/jmrs.208 PMC5587663

[8] Halkett G , Merchant S , Jiwa M , et al. Effective communication and information provision in radiotherapy—the role of radiation therapists. J Radiother Pract 2010; 9: 3–16.

[9] Halkett G , O'Connor M , Jefford M , et al. RT Prepare: a radiation therapist‐delivered intervention reduces psychological distress in women with breast cancer referred for radiotherapy. Br J Cancer 2018; 118: 1549–58. 29855611 10.1038/s41416-018-0112-z PMC6008448

[10] Halkett G , O'Connor M , Aranda S , et al. Communication skills training for RTs. J Med Radiat Sci. 2016; 63: 232–41. 27741388 10.1002/jmrs.171 PMC5167288

[11] Youens D , Halkett G , Wright C , et al. Assessing the Cost‐Effectiveness of RT Prepare: A radiation therapist‐delivered intervention for reducing psychological distress prior to radiotherapy. Psychooncology 2019; 28: 1110–8. 30884030 10.1002/pon.5065

[12] Elsner KL , Naehrig DN , Halkett GKB , Dhillon HM . How do radiation therapists detect and manage patients experiencing anxiety in the radiation oncology setting? A vignette study. Support Care Cancer 2021; 29: 5973–81. 33770258 10.1007/s00520-021-06133-9

[13] Butow P , Shepherd HL , Cuddy J , et al. Acceptability and appropriateness of a clinical pathway for managing anxiety and depression in cancer patients: a mixed methods study of staff perspectives. BMC Health Serv Res 2021; 21: 1243. 34789239 10.1186/s12913-021-07252-z PMC8600707

[14] Butow P , Shepherd HL , Cuddy J , et al. Staff perspectives on the feasibility of a clinical pathway for anxiety and depression in cancer care, and mid‐implementation adaptations. BMC Health Serv Res 2022; 22: 192. 35164772 10.1186/s12913-022-07532-2 PMC8842573

[15] Girgis A , Durcinoska I , Arnold A , et al. Web‐Based Patient‐Reported Outcome Measures for Personalized Treatment and Care (PROMPT‐Care): Multicenter Pragmatic Nonrandomized Trial. J Med Internet Res 2020; 22: e19685. 33118954 10.2196/19685 PMC7661255

[16] Arnold BL , Girgis PA , Dhillon AH , Descallar J , Halkett AG . Does a communication skills program enable symptom and distress screening?: The impact of training on radiation therapists' confidence, knowledge, and use of distress screening. J Med Imaging Radiat Sci 2021; 52: 399–408. 34183301 10.1016/j.jmir.2021.05.011

[17] Arnold BL , Halkett G , Dhillon H , Girgis A . Do radiation therapists feel able to routinely screen for symptoms and distress in people with cancer: barriers impacting practice. J Med Radiat Sci. 2021; 68: 149–56. 33729701 10.1002/jmrs.465 PMC8168062

[18] Wang LJ , Casto B , Luh JY , Wang SJ . Virtual reality‐based education for patients undergoing radiation therapy. J Cancer Educ 2022; 37: 694–700. 32970303 10.1007/s13187-020-01870-7 PMC7512212

[19] Johnson K , Liszewski B , Dawdy K , Lai Y , McGuffin M . Learning in 360 degrees: A pilot study on the use of virtual reality for radiation therapy patient education. J Med Imaging Radiat Sci. 2020; 51: 221–6. 32046946 10.1016/j.jmir.2019.12.008

[20] Keall P , University of Sydney, Western Sydney Local Health District . Head and Neck Cancer|Radiation Therapy Complication|Anxiety ClinicalTrials.gov [Internet]: National Library of Medicine. 2020. Available from: https://ClinicalTrials.gov/show/NCT04266223.

